# Effect of Amino Acids and Sodium Chloride on d-Sorbitol in Aqueous Solutions at Different Temperatures: Volumetric and Acoustic Approach

**DOI:** 10.1007/s10953-018-0820-2

**Published:** 2018-10-20

**Authors:** Dorota Warmińska

**Affiliations:** Department of Physical Chemistry, Chemical Faculty, Gdańsk University of Technology, Narutowicza 11/12, 80-233 Gdańsk, Poland

**Keywords:** Density, Sound velocity, d-Sorbitol, Aqueous amino acid solutions, Sodium chloride

## Abstract

Apparent molar volumes and apparent molar compressibilities for d-sorbitol in (0.05, 0.1, 0.2 and 0.3) mol·kg^−1^ aqueous solutions of l-alanine, l-cysteine and l-histidine and NaCl have been determined from measurements of solution density at *T* = (288.15, 298.15, 308.15 and 318.15) K and sound velocity at *T* = 298.15 K, as a function of the concentration of the sugar alcohol. The data were used to obtain the limiting apparent molar volumes, limiting apparent molar compressibilities and the corresponding transfer parameters. Limiting apparent molar expansibilities and their second order derivatives and volume interaction coefficients were also estimated. These parameters are discussed in terms of d-sorbitol and co-solute (amino acid or sodium chloride) interactions in aqueous solutions.

## Introduction

In recent years, sugar alcohols, owing to their desirable properties, have found applications in many branches of industry. Properties of polyols, such as good taste, low calorie content and little effect on blood sugar levels have paved the way for the use of these compounds in the production of prepared foods, cosmetics and pharmaceuticals [1–3]. It has been established that polyols enable stabilization of the native state of proteins and affect their denaturalization, solubility and folding/unfolding behavior [4, 5]. Thus, knowledge of the properties of polyols in aqueous solutions of amino acids is essential for understanding the chemistry of biological systems.

Among various polyols, d-sorbitol, a six-carbon sugar alcohol, is widely used as sweetening agent, plasticizer, as well as capsule and tablet diluent [6, 7]. It is known that d-sorbitol, despite having a structure similar to that of its isomer d-mannitol, shows a real difference in solubility in water and in osmotic pressure coefficients [8]. It has also been suggested that d-sorbitol is a relatively stronger disruptor of the water structure than d-mannitol [9].

To the best of our knowledge, reports on the effects of amino acids on the structure of water modified by sugar alcohol are still scare. Most of the thermodynamic data have been collected for systems with d-mannitol and xylitol [10–15]. So far, few studies have focused on the volumetric and acoustic properties of aqueous solutions containing d-sorbitol and amino acids. Density and sound velocity data at 298.15 K for some ternary mixtures (amino acid + d-sorbitol + water) have been reported by Jha and Kishore [16]. Moreover, Ren et al. published volumetric data for such systems for a wider range of temperatures [17]. The authors of both publications calculated the limiting molar volumes of transfer from water to aqueous d-sorbitol solutions for amino acids and they postulated the existence of hydrophilic/polar group interactions in the systems. The same conclusion was reached by Banipal et al. who studied volumetric properties of d-sorbitol in aqueous solutions of l-glycine [18].

The present study was aimed at providing some additional data on the properties of d-sorbitol in aqueous amino acid solutions using volumetric and acoustic measurements. The densities at *T *= (288.15, 298.15, 308.15 and 318.15) K and sound velocities at *T* = 298.15 K for d-sorbitol in (0.05, 0.1, 0.2 and 0.3) mol·kg^−1^ aqueous solutions of l-alanine, l-cysteine and l-histidine are reported. Additionally, corresponding data for d-sorbitol in aqueous solutions of NaCl was also collected. The paper presents calculations of the apparent molar volumes and the apparent molar compressibilities, as well as their limiting values. The corresponding transfer parameters and limiting apparent molar expansibilities are also estimated. The evaluated parameters were correlated and interpreted in terms of the different types of interactions that can occur in ternary systems. The structure making/breaking tendency of d-sorbitol in aqueous amino acid or NaCl solutions is also analyzed.

## Experimental

### Chemical Used

**Fig. 1:**
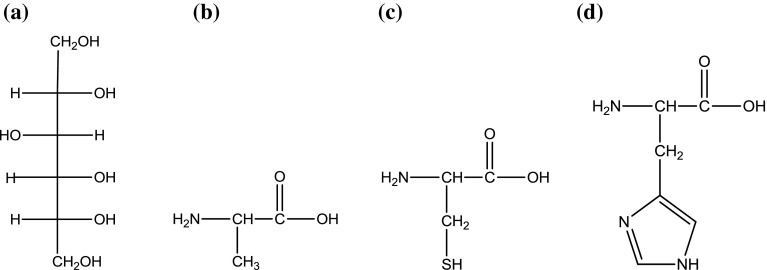
Molecular structures of d-sorbitol (**a**), l-alanine (**b**), l-cysteine (**c**) and l-histidine (**d**)

d-sorbitol (≥ 0.98), l-alanine (≥ 0.99), l-cysteine (≥ 0.99) and l-histidine (≥ 0.99) were obtained from Carl Roth GMbH + Co. KG, sodium chloride (≥ 0.999) was purchased from POCh and were used without further purification. Table 1 briefly describes the properties of the chemicals and Fig. 1 shows their structures. Before measurements, d-sorbitol and all amino acids and NaCl were dried under reduced pressure at 323 K. Deionized, doubly distilled, degassed water with a specific conductance of 1.15 × 10^−6^ S·cm^−1^ was used for the preparation of (0.05, 0.1, 0.2 and 0.3) mol·kg^−1^ solutions of l-alanine and l-cysteine, whereas in the case of l-histidine only three solutions (0.05, 0.1 and 0.2) mol·kg^−1^ were made due to its lower solubility. All the solutions were prepared by weight dilution of the stock solution of d-sorbitol using a Mettler Toledo balance with a precision of ± 0.0001 g.

**Table 1:** Provenance and mass fraction purity of the compounds studied

Chemical name	Source	CAS number	Mass fraction purity
d-Sorbitol	Carl Roth GMbH + Co. KG	50-70-4	≥ 0.98^a^
l-Alanine	Carl Roth GMbH + Co. KG	56-41-7	≥ 0.99^a^
l-Cysteine	Carl Roth GMbH + Co. KG	52-90-4	≥ 0.99^a^
l-Histidine	Carl Roth GMbH + Co. KG	71-00-1	≥ 0.99^a^
NaCl	POCh	7647-14-5	≥ 0.999^a^

^a^As stated by the supplier. The chemicals were used as such without further purification

### Apparatus and Procedure

The densities of the ternary mixtures were measured at different temperatures using a digital vibrating-tube analyzer (Anton Paar DMA 5000, Austria), equipped with a built-in solid-state thermostat that controls the temperature by means of a combination of thermoelectric Peltier elements and an intergrated Pt-100 resistance thermometer with an accuracy of 0.01 K. The measurement cell in the apparatus is made of borosilicate glass. Prior to each series of measurements, the apparatus was calibrated using doubly distilled and degassed water and with dry air at atmospheric pressure (0.1 MPa). The standard uncertainty in the density measurements was within ± 35 × 10^−3^ kg·m^−3^.

The sound velocities were determined with a standard uncertainty of 0.15 m·s^−1^ using the sound analyzer OPTIME 1.0 from OPTEL (Poland), based on the time of flight method. Measurements are based on the determination of the time that the acoustic signal with a frequency of 8 MHz takes to pass through a sample of known length. The length of the quartz measurement cell was determined with doubly distilled water, with the value 1496.69 m·s^−1^ used as the sound velocity in pure water at 298.15 K. Temperature during measurements was stabilized by a constant temperature bath (model: PolyScience 8202) at 298.15 ± 0.01 K.

## Results and Discussion

### Volumetric Properties

The experimental values of molality, density and apparent molar volume at various temperatures of the solutions of d-sorbitol in aqueous l-alanine, l-cysteine, l-histidine and NaCl are reported in Tables 2, 3, 4 and 5. The results show that density increases with an increase in the concentration of d-sorbitol and also with the molality of amino acids or sodium chloride. Irrespective of temperature, the increase in density resulting from increased concentration of d-sorbitol is greatest in the case of solutions in water. The greater the amount of amino acid or NaCl in the solution, the smaller is the increase. Moreover, with increasing temperature the observed effect, i.e. the increase in density with increasing concentration of d-sorbitol, becomes less pronounced. For all the systems studied, the density decreases with an increase in temperature.

**Table 2:** The densities and apparent molar volumes of d-sorbitol in l-alanine aqueous solutions at temperature *T* = 288.15, 298.15, 308.15 and 318.15 K

*m* (mol·kg^−1^)	288.15 K		298.15 K		308.15 K		318.15 K	
	*d* (kg·m^−3^)	10^6^·*ϕ*_V_ (m^3^·mol^−1^)	*d* (kg·m^−3^)	10^6^·*ϕ*_V_ (m^3^·mol^−1^)	*d* (kg·m^−3^)	10^6^·*ϕ*_V_ (m^3^·mol^−1^)	*d* (kg·m^−3^)	10^6^·*ϕ*_V_ (m^3^·mol^−1^)
Water								
0.00000	999.120	–	997.055	–	994.041	–	990.217	–
0.04020	1001.712	117.43	999.600	118.72	996.548	119.84	992.692	120.87
0.06324	1003.186	117.45	1001.047	118.74	997.973	119.87	994.100	120.88
0.08443	1004.533	117.47	1002.370	118.75	999.275	119.89	995.386	120.90
0.09748	1005.359	117.48	1003.180	118.77	1000.074	119.90	996.175	120.91
0.1273	1007.239	117.50	1005.024	118.80	1001.890	119.93	997.970	120.92
0.1472	1008.481	117.52	1006.245	118.81	1003.093	119.93	999.155	120.95
0.1741	1010.152	117.56	1007.887	118.84	1004.709	119.97	1000.756	120.95
0.1986	1011.662	117.59	1009.371	118.86	1006.173	119.98	1002.199	120.98
0.2368	1014.003	117.63	1011.668	118.90	1008.438	120.00	1004.437	121.00
0.2782	1016.506	117.67	1014.125	118.94	1010.858	120.05	1006.830	121.03
0.3167	1018.810	117.72	1016.392	118.98	1013.090	120.08	1009.039	121.05
0.05 mol·kg^−1^								
0.0000	1000.568	–	998.476	–	995.450	–	991.615	–
0.04812	1003.661	117.50	1001.514	118.76	998.442	119.90	994.570	120.90
0.06101	1004.482	117.52	1002.32	118.79	999.236	119.92	995.355	120.91
0.07938	1005.647	117.56	1003.465	118.81	1000.363	119.95	996.469	120.93
0.09376	1006.556	117.57	1004.358	118.82	1001.243	119.95	997.338	120.94
0.1207	1008.252	117.60	1006.023	118.86	1002.884	119.98	998.959	120.96
0.1581	1010.577	117.64	1008.307	118.89	1005.134	120.01	1001.183	120.99
0.1722	1011.455	117.64	1009.168	118.90	1005.981	120.03	1002.020	121.00
0.1922	1012.685	117.69	1010.378	118.93	1007.174	120.05	1003.200	121.01
0.2250	1014.690	117.71	1012.348	118.96	1009.112	120.08	1005.118	121.03
0.2671	1017.238	117.77	1014.856	118.99	1011.579	120.12	1007.557	121.07
0.3042	1019.459	117.81	1017.036	119.03	995.450	120.16	1009.686	121.09
0.1 mol·kg^−1^								
0.0000	1002.079	–	999.956	–	996.911	–	993.050	–
0.03207	1004.135	117.67	1001.975	118.978	998.901	120.06	995.017	121.01
0.05503	1005.598	117.70	1003.411	118.98	1000.315	120.09	996.415	121.03
0.07659	1006.961	117.74	1004.750	119.01	1001.634	120.11	997.719	121.06
0.09114	1007.878	117.75	1005.650	119.02	1002.521	120.13	998.596	121.07
0.1165	1009.467	117.77	1007.209	119.05	1004.057	120.15	1000.115	121.09
0.1514	1011.636	117.80	1009.339	119.08	1006.155	120.18	1002.189	121.12
0.1655	1012.504	117.82	1010.192	119.09	1006.996	120.19	1003.020	121.13
0.1845	1013.675	117.84	1011.342	119.11	1008.128	120.21	1004.139	121.15
0.2168	1015.649	117.87	1013.278	119.14	1010.035	120.24	1006.024	121.18
0.2571	1018.089	117.90	1015.670	119.19	1012.394	120.28	1008.353	121.23
0.2932	1020.250	117.95	1017.795	119.23	1014.482	120.32	1010.420	121.26
0.2 mol·kg^−1^								
0.0000	1004.877	–	1002.700	–	999.608	–	995.733	–
0.04673	1007.856	117.81	1005.625	119.08	1002.492	120.13	998.584	121.07
0.05916	1008.642	117.82	1006.397	119.09	1003.253	120.14	999.337	121.07
0.07854	1009.863	117.85	1007.595	119.12	1004.434	120.18	1000.505	121.09
0.09352	1010.802	117.83	1008.517	119.11	1005.343	120.17	1001.405	121.09
0.1198	1012.44	117.87	1010.126	119.14	1006.928	120.20	1002.973	121.11
0.1560	1014.679	117.90	1012.324	119.16	1009.096	120.22	1005.115	121.14
0.1661	1015.300	117.91	1012.932	119.18	1009.696	120.23	1005.710	121.14
0.1886	1016.679	117.94	1014.289	119.19	1011.032	120.25	1007.032	121.16
0.2203	1018.608	117.95	1016.181	119.22	1012.897	120.28	1008.875	121.19
0.2629	1021.167	118.01	1018.700	119.25	1015.382	120.30	1011.327	121.23
0.2997	1023.368	118.03	1020.852	119.29	1017.506	120.33	1013.429	121.26
0.3 mol·kg^−1^								
0.0000	1007.413	–	1005.196	–	1002.069	–	998.179	–
0.04969	1010.565	117.96	1008.293	119.18	1005.120	120.29	1001.197	121.18
0.06298	1011.400	117.98	1009.114	119.19	1005.929	120.29	1001.997	121.19
0.07810	1012.348	117.99	1010.046	119.20	1006.846	120.31	1002.905	121.20
0.09708	1013.533	118.00	1011.209	119.22	1007.993	120.32	1004.039	121.21
0.1264	1015.350	118.04	1012.996	119.24	1009.752	120.35	1005.780	121.23
0.1621	1017.542	118.07	1015.150	119.28	1011.874	120.38	1007.877	121.27
0.1747	1018.313	118.06	1015.906	119.26	1012.620	120.37	1008.617	121.27
0.1977	1019.714	118.10	1017.282	119.31	1013.975	120.41	1009.958	121.29
0.2320	1021.782	118.12	1019.312	119.34	1015.974	120.44	1011.938	121.31
0.2764	1024.439	118.15	1021.922	119.36	1018.546	120.46	1014.475	121.36
0.3149	1026.710	118.19	1024.155	119.40	1020.742	120.50	1016.652	121.38

Standard uncertainties u are u(*T*) = 0.01 K, u(*d*) = 0.035 kg·m^−3^, standard uncertainty of molality u(*m*) = 0.001 mol·kg^−1^ and standard uncertainty of experimental pressure u(*p*) = 10 kPa

**Table 3:** The densities and apparent molar volumes of d-sorbitol in l-cysteine aqueous solutions at temperature *T* = 288.15, 298.15, 308.15 and 318.15 K

*m* (mol·kg^−1^)	288.15 K		298.15 K		308.15 K		318.15 K	
	*d* (kg·m^−3^)	10^6^·*ϕ*_V_ (m^3^·mol^−1^)	*d* (kg·m^−3^)	10^6^·*ϕ*_V_ (m^3^·mol^−1^)	*d* (kg·m^−3^)	10^6^·*ϕ*_V_ (m^3^·mol^−1^)	*d* (kg·m^−3^)	10^6^·*ϕ*_V_ (m^3^·mol^−1^)
0.05 mol·kg^−1^								
0.00000	1001.508	–	999.414	–	996.362	–	992.521	–
0.06327	1005.560	117.57	1003.389	118.90	1000.279	120.00	996.391	120.98
0.07674	1006.414	117.58	1004.227	118.92	1001.105	120.01	997.207	120.99
0.09873	1007.802	117.60	1005.588	118.94	1002.447	120.02	998.533	121.00
0.1259	1009.508	117.62	1007.261	118.96	1004.097	120.04	1000.163	121.01
0.1630	1011.813	117.66	1009.523	118.99	1006.326	120.07	1002.366	121.04
0.1783	1012.759	117.68	1010.451	119.00	1007.240	120.08	1003.268	121.06
0.1993	1014.051	117.69	1011.716	119.03	1008.490	120.09	1004.504	121.06
0.2327	1016.087	117.72	1013.712	119.06	1010.459	120.11	1006.445	121.10
0.2782	1018.838	117.76	1016.412	119.08	1013.116	120.16	1009.073	121.13
0.3166	1021.130	117.79	1018.659	119.12	1015.330	120.19	1011.265	121.15
0.1 mol·kg^−1^								
0.0000	1003.922	–	1001.772	–	998.688	–	994.815	–
0.05122	1007.191	117.76	1004.982	119.02	1001.853	120.08	997.943	121.04
0.06342	1007.963	117.78	1005.740	119.04	1002.600	120.11	998.682	121.04
0.08443	1009.287	117.77	1007.041	119.04	1003.882	120.10	999.950	121.04
0.1004	1010.290	117.79	1008.025	119.06	1004.852	120.12	1000.909	121.06
0.1252	1011.835	117.81	1009.542	119.07	1006.348	120.13	1002.386	121.08
0.1621	1014.121	117.84	1011.786	119.11	1008.561	120.16	1004.575	121.10
0.1762	1014.988	117.85	1012.638	119.11	1009.400	120.17	1005.405	121.11
0.2009	1016.498	117.87	1014.120	119.13	1010.862	120.19	1006.848	121.13
0.2349	1018.564	117.88	1016.144	119.16	1012.858	120.21	1008.823	121.15
0.2836	1021.489	117.95	1019.023	119.19	1015.695	120.25	1011.625	121.19
0.3195	1023.622	117.97	1021.118	119.22	1017.758	120.28	1013.667	121.21
0.2 mol·kg^−1^								
0.0000	1008.617	–	1006.381	–	1003.240	–	999.317	–
0.06330	1012.626	117.89	1010.319	119.12	1007.122	120.18	1003.154	121.13
0.08324	1013.875	117.91	1011.546	119.14	1008.332	120.20	1004.350	121.14
0.1016	1015.024	117.91	1012.674	119.15	1009.444	120.20	1005.449	121.15
0.1205	1016.195	117.93	1013.825	119.16	1010.578	120.22	1006.570	121.16
0.1574	1018.470	117.95	1016.058	119.19	1012.78	120.24	1008.746	121.19
0.1928	1020.627	117.98	1018.178	119.20	1014.868	120.27	1010.810	121.21
0.2258	1022.626	118.00	1020.140	119.23	1016.802	120.29	1012.724	121.22
0.2680	1025.149	118.05	1022.622	119.26	1019.250	120.32	1015.142	121.25
0.3065	1027.436	118.07	1024.864	119.29	1003.240	120.35	1017.326	121.28
0.3 mol·kg^−1^								
0.0000	1013.226	–	1010.912	–	1007.706	–	1003.741	–
0.04672	1016.172	118.03	1013.805	119.27	1010.558	120.32	1006.563	121.26
0.05997	1017.001	118.04	1014.619	119.28	1011.361	120.32	1007.353	121.27
0.07901	1018.187	118.05	1015.784	119.29	1012.509	120.34	1008.488	121.28
0.09337	1019.079	118.05	1016.66	119.29	1013.372	120.34	1009.341	121.29
0.1202	1020.732	118.08	1018.284	119.31	1014.973	120.36	1010.923	121.31
0.1550	1022.865	118.11	1020.38	119.33	1017.04	120.37	1012.965	121.32
0.1693	1023.737	118.11	1021.234	119.34	1017.882	120.39	1013.799	121.32
0.1891	1024.932	118.13	1022.409	119.35	1019.043	120.40	1014.943	121.34
0.2224	1026.936	118.14	1024.375	119.38	1020.976	120.43	1016.859	121.36
0.2623	1029.313	118.18	1026.713	119.40	1023.277	120.45	1019.132	121.39
0.3013	1031.608	118.22	1028.969	119.42	1025.506	120.47	1021.332	121.41

Standard uncertainties u are u(*T*) = 0.01 K, u(*d*) = 0.035 kg·m^−3^, standard uncertainty of molality u(*m*) = 0.001 mol·kg^−1^ and standard uncertainty of experimental pressure u(*p*) = 10 kPa

**Table 4:** The densities and apparent molar volumes of d-sorbitol in L-histidine aqueous solutions at temperature *T* = 288.15, 298.15, 308.15 and 318.15 K

*m* (mol·kg^−1^)	288.15 K		298.15 K		308.15 K		318.15 K	
	*d* (kg·m^−3^)	10^6^·*ϕ*_V_ (m^3^·mol^−1^)	*d* (kg·m^−3^)	10^6^·*ϕ*_V_ (m^3^·mol^−1^)	*d* (kg·m^−3^)	10^6^·*ϕ*_V_ (m^3^·mol^−1^)	*d* (kg·m^−3^)	10^6^·*ϕ*_V_ (m^3^·mol^−1^)
0.05 mol·kg^−1^								
0.0000	1001.985	–	999.855	–	996.790	–	992.924	–
0.05323	1005.394	117.62	1003.202	118.905	1000.088	120.006	996.184	120.955
0.06586	1006.196	117.63	1003.989	118.921	1000.864	120.013	996.951	120.962
0.07846	1006.994	117.64	1004.773	118.918	1001.636	120.017	997.714	120.967
0.09625	1008.115	117.66	1005.873	118.941	1002.720	120.038	998.787	120.972
0.1229	1009.789	117.68	1007.517	118.955	1004.341	120.042	1000.389	120.981
0.1551	1011.785	117.71	1009.479	118.971	1006.273	120.067	1002.300	120.998
0.1705	1012.740	117.72	1010.415	118.988	1007.196	120.079	1003.213	121.017
0.1935	1014.155	117.74	1011.807	118.998	1008.567	120.092	1004.569	121.017
0.2235	1015.987	117.77	1013.605	119.026	1010.340	120.111	1006.323	121.032
0.2630	1018.376	117.81	1015.952	119.057	1012.654	120.137	1008.612	121.053
0.3063	1020.972	117.84	1018.500	119.089	1015.164	120.171	1011.098	121.072
0.1 mol·kg^−1^								
0.0000	1004.806	–	1002.615	–	999.505	–	995.613	–
0.04773	1007.849	117.80	1005.604	119.05	1002.452	120.11	998.525	121.08
0.06064	1008.665	117.82	1006.406	119.05	1003.243	120.11	999.306	121.09
0.07851	1009.791	117.82	1007.512	119.06	1004.334	120.11	1000.384	121.09
0.09436	1010.785	117.84	1008.489	119.07	1005.297	120.12	1001.335	121.10
0.1271	1012.826	117.87	1010.495	119.09	1007.275	120.14	1003.290	121.11
0.1682	1015.367	117.89	1012.990	119.12	1009.735	120.17	1005.720	121.14
0.1910	1016.760	117.90	1014.358	119.13	1011.084	120.18	1007.053	121.15
0.2240	1018.770	117.92	1016.333	119.15	1013.030	120.20	1008.977	121.17
0.2673	1021.370	117.97	1018.890	119.19	1015.552	120.23	1011.470	121.19
0.3038	1023.546	118.00	1021.028	119.21	1017.660	120.25	1013.554	121.21
0.2 mol·kg^−1^								
0.0000	1010.315	–	1008.006	–	1004.808	–	1000.852	–
0.04743	1013.314	118.02	1010.952	119.25	1007.713	120.29	1003.724	121.22
0.06299	1014.290	118.03	1011.911	119.25	1008.658	120.30	1004.659	121.22
0.0837	1015.583	118.04	1013.182	119.26	1009.911	120.31	1005.897	121.24
0.09852	1016.504	118.04	1014.086	119.27	1010.802	120.32	1006.779	121.24
0.1254	1018.168	118.05	1015.720	119.28	1012.413	120.33	1008.372	121.25
0.1636	1020.511	118.08	1018.023	119.29	1014.683	120.35	1010.615	121.27
0.1784	1021.410	118.08	1018.904	119.31	1015.554	120.35	1011.475	121.28
0.1992	1022.671	118.10	1020.144	119.31	1016.775	120.36	1012.682	121.29
0.2339	1024.762	118.11	1022.198	119.34	1018.800	120.38	1014.685	121.31
0.2783	1027.407	118.14	1024.795	119.36	1021.359	120.41	1017.217	121.32
0.3175	1029.724	118.15	1027.069	119.38	1023.600	120.43	1019.435	121.34

Standard uncertainties u are u(*T*) = 0.01 K, u(*d*) = 0.035 kg·m^−3^, standard uncertainty of molality u(*m*) = 0.001 mol·kg^−1^ and standard uncertainty of experimental pressure u(*p*) = 10 kPa

**Table 5:** The densities and apparent molar volumes of d-sorbitol in NaCl aqueous solutions at temperature *T* = 288.15, 298.15, 308.15 and 318.15 K

*m* (mol·kg^−1^)	288.15 K		298.15 K		308.15 K		318.15 K	
	*d* (kg·m^−3^)	10^6^·*ϕ*_V_ (m^3^·mol^−1^)	*d* (kg·m^−3^)	10^6^·*ϕ*_V_ (m^3^·mol^−1^)	*d* (kg·m^−3^)	10^6^·*ϕ*_V_ (m^3^·mol^−1^)	*d* (kg·m^−3^)	10^6^·*ϕ*_V_ (m^3^·mol^−1^)
0.05 mol·kg^−1^								
0.00000	1001.242	–	999.128	–	996.069	–	992.223	–
0.05128	1004.527	117.65	1002.352	118.96	999.246	120.06	995.362	121.04
0.06540	1005.424	117.67	1003.233	118.96	1000.114	120.06	996.219	121.05
0.08638	1006.751	117.68	1004.535	118.98	1001.397	120.08	997.487	121.06
0.1002	1007.623	117.69	1005.391	118.99	1002.240	120.09	998.320	121.07
0.1310	1009.552	117.71	1007.283	119.01	1004.106	120.10	1000.163	121.08
0.1684	1011.872	117.74	1009.561	119.04	1006.352	120.12	1002.382	121.10
0.1844	1012.858	117.76	1010.530	119.05	1007.306	120.13	1003.325	121.11
0.2059	1014.177	117.78	1011.825	119.06	1008.582	120.14	1004.585	121.12
0.2412	1016.323	117.81	1013.932	119.09	1010.659	120.17	1006.639	121.14
0.2876	1019.115	117.86	1016.674	119.13	1013.364	120.20	1009.312	121.16
0.3277	1021.506	117.88	1019.018	119.16	996.069	120.22	1011.599	121.18
0.1 mol·kg^−1^								
0.0000	1003.240	–	1001.069	–	997.976	–	994.104	–
0.04895	1006.364	117.81	1004.137	119.08	1001.001	120.14	997.093	121.10
0.06382	1007.306	117.81	1005.061	119.07	1001.912	120.13	997.994	121.10
0.08004	1008.329	117.81	1006.065	119.09	1002.902	120.15	998.972	121.11
0.09695	1009.391	117.82	1007.108	119.10	1003.931	120.15	999.988	121.12
0.1249	1011.138	117.84	1008.824	119.11	1005.623	120.16	1001.66	121.13
0.1578	1013.173	117.86	1010.821	119.13	1007.592	120.18	1003.606	121.15
0.1759	1014.289	117.87	1011.917	119.14	1008.672	120.19	1004.673	121.16
0.1956	1015.498	117.88	1013.105	119.14	1009.841	120.21	1005.831	121.16
0.2301	1017.600	117.90	1015.168	119.16	1011.876	120.22	1007.842	121.17
0.2616	1019.500	117.91	1017.032	119.18	1013.713	120.24	1009.659	121.18
0.3121	1022.520	117.94	1019.995	119.21	997.976	120.26	1012.548	121.20
0.2 mol·kg^−1^								
0.0000	1007.413	–	1005.129	–	1001.967	–	998.046	–
0.05714	1011.025	118.12	1008.680	119.30	1005.469	120.34	1001.507	121.30
0.07229	1011.974	118.13	1009.613	119.31	1006.389	120.35	1002.417	121.30
0.09448	1013.358	118.14	1010.974	119.31	1007.731	120.36	1003.743	121.30
0.1129	1014.500	118.145	1012.097	119.32	1008.839	120.36	1004.838	121.31
0.1454	1016.502	118.16	1014.065	119.34	1010.780	120.37	1006.756	121.32
0.1860	1018.984	118.17	1016.504	119.35	1013.184	120.39	1009.133	121.33
0.2041	1020.084	118.18	1017.587	119.36	1014.251	120.40	1010.188	121.34
0.2276	1021.500	118.19	1018.978	119.37	1015.624	120.41	1011.544	121.35
0.2675	1023.89	118.21	1021.326	119.39	1017.939	120.42	1013.832	121.37
0.3184	1026.899	118.24	1024.285	119.42	1020.857	120.45	1016.716	121.39
0.3635	1029.540	118.26	1026.880	119.44	1023.417	120.46	1019.245	121.41
0.3 mol·kg^−1^								
0.0000	1011.551	–	1009.171	–	1005.934	–	1001.962	–
0.06543	1015.653	118.35	1013.203	119.53	1009.912	120.54	1005.895	121.47
0.08987	1017.167	118.36	1014.691	119.55	1011.380	120.56	1007.347	121.47
0.1059	1018.155	118.37	1015.662	119.56	1012.339	120.55	1008.295	121.47
0.1407	1020.291	118.38	1017.762	119.57	1014.410	120.57	1010.343	121.49
0.1736	1022.290	118.39	1019.725	119.58	1016.348	120.58	1012.258	121.50
0.1936	1023.491	118.41	1020.907	119.59	1017.514	120.59	1013.411	121.51
0.2162	1024.850	118.42	1022.243	119.60	1018.830	120.60	1014.714	121.51
0.2511	1026.933	118.43	1024.290	119.61	1020.850	120.61	1016.712	121.52
0.2983	1029.720	118.45	1027.030	119.62	1023.552	120.63	1019.383	121.54
0.3448	1032.430	118.47	1029.692	119.65	1026.180	120.64	1021.980	121.56

Standard uncertainties u are u(*T*) = 0.01 K, u(*d*) = 0.035 kg·m^−3^, standard uncertainty of molality u(*m*) = 0.001 mol·kg^−1^ and standard uncertainty of experimental pressure u(*p*) = 10 kPa

The corresponding values of the apparent molar volumes *ϕ*_V_ of d-sorbitol in the ternary systems were calculated from the densities of the solutions using the following equation:

1 $$\phi_{\text{V}} = \frac{{\left( {d_{0} - d} \right)}}{{mdd_{0} }} + \frac{{M_{2} }}{d}$$

where *m* denotes the molality of solution, *d* and *d*_0_ are densities of solution and solvent, respectively, and *M*_2_ is the molar mass of the solute.

**Fig. 2:**
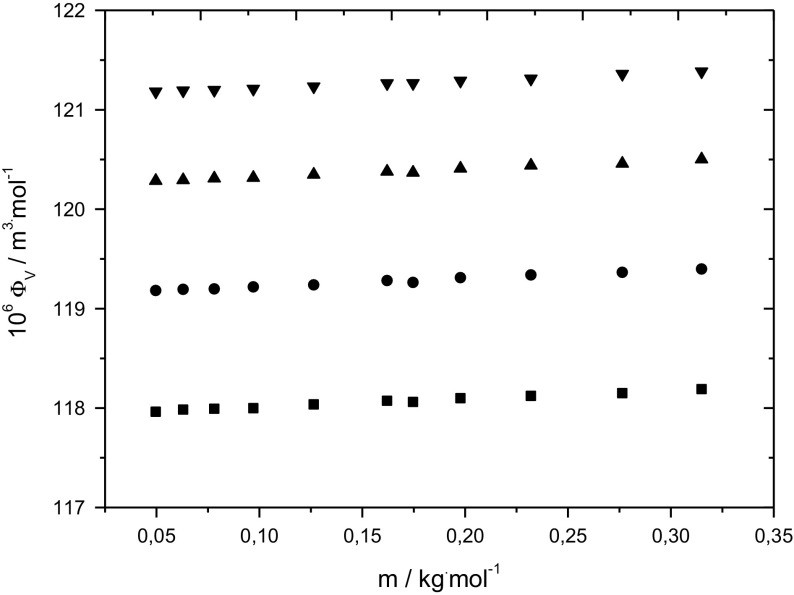
The concentration dependencies of the apparent molar volume of d-sorbitol in 0.3 mol·kg^−1^ l-alanine at temperatures between 288.15 and 318.15 K: *T* = 288.15 K (filled squares), *T* = 298.15 K (filled circle), *T* = 308.15 K (filled triangle) and *T* = 318.15 K (filled inverted triangle)

Figure 2 presents, as an example, the concentration dependencies of the apparent molar volumes of d-sorbitol in 0.3 mol·kg^−1^ l-alanine at temperatures between 288.15 and 318.15 K. As seen from the plots, the relationship is linear across the whole d-sorbitol molality range and the whole temperature range studied. Therefore, the limiting apparent molar volumes $\phi_{\text{V}}^{0}$ were evaluated by extrapolating the respective plots to infinite dilution using the equation:

2 $$\phi_{\text{V}} = \phi_{\text{V}}^{0} + S_{\text{V}} m$$

The parameters of Eq. 2 and their standard deviations $\sigma$ are given in Tables 6, 7, 8, and 9. Table 6 includes literature values of the limiting apparent molar volumes of d-sorbitol in water [18–20]. As is seen, the data from the present study shows good agreement with those in the literature. Almost no data for $\phi_{\text{V}}^{0}$ in the presence of NaCl or amino acids are available for comparison. Exceptions include volumetric data for d-sorbitol in aqueous 2 and 4 mol·kg^−1^ NaCl at 298.15 K reported by Jasra and Ahluwalia and the data for (l-alanine + sorbitol + water) ternary solutions obtained by Jha and Kishore and by Ren et al. [16, 17, 21]. However, the authors measured the densities of l-alanine in fixed concentrations of aqueous d-sorbitol, not d-sorbitol in fixed concentrations of aqueous l-alanine as in the present study. Moreover, different temperature ranges and distinctly higher concentrations of d-sorbitol were studied. Thus, direct comparison of the results is impossible.

**Table 6:** The coefficients of Eq. 2 with the corresponding residual standard deviations $\sigma$ for d-sorbitol in l-alanine aqueous solutions at temperatures *T* = 288.15, 298.15, 308.15 and 318.15 K

*T* (*K*)	10^6^·$\phi_{\text{V}}^{0}$ (m^3^·mol^−1^)	10^6^*S*_V_ (m^3^·kg·mol^−2^)	10^6^σ (m^3^·mol^−1^)
Water			
288.15	117.38 ± 0.007 (116.94^a^, 116.88^b^, 117.82^c^)	1.06 ± 0.034	0.009
298.15	118.68 ± 0.005 (118.62^a^,118.97^b^. 119.15^c^)	0.94 ± 0.020	0.004
308.15	119.82 ± 0.007 (119.95^a^, 119.68^b^)	0.83 ± 0.028	0.006
318.15	120.84 ± 0.008 (121.13^a^, 121.06^b^)	0.66 ± 0.022	0.005
0.05 l-alanine			
288.15	117.46 ± 0.009	1.17 ± 0.030	0.007
298.15	118.73 ± 0.006	1.01 ± 0.032	0.007
308.15	119.86 ± 0.005	0.97 ± 0.025	0.006
318.15	120.87 ± 0.004	0.74 ± 0.018	0.004
0.1 l-alanine			
288.15	117.65 ± 0.008	1.00 ± 0.025	0.006
298.15	118.93 ± 0.008	0.99 ± 0.031	0.007
308.15	120.00 ± 0.008	0.97 ± 0.022	0.005
318.15	120.98 ± 0.004	0.95 ± 0.020	0.005
0.2 l-alanine			
288.15	117.76 ± 0.008	0.89 ± 0.024	0.006
298.15	119.04 ± 0.004	0.81 ± 0.021	0.005
308.15	120.10 ± 0.008	0.77 ± 0.022	0.006
318.15	121.03 ± 0.009	0.75 ± 0.031	0.007
0.3 l-alanine			
288.15	117.93 ± 0.008	0.84 ± 0.024	0.005
298.15	119.14 ± 0.006	0.83 ± 0.023	0.006
308.15	120.24 ± 0.007	0.81 ± 0.022	0.006
318.15	121.14 ± 0.004	0.77 ± 0.021	0.005

^a^Ref. [18]

^b^Ref. [19]

^c^Ref. [20]

**Table 7:** The coefficients of Eq. 2 with the corresponding residual standard deviations $\sigma$ for d-sorbitol in l-cysteine aqueous solution at temperature *T* = 288.15, 298.15, 308.15 and 318.15 K

*T*/K	10^6^·$\phi_{\text{V}}^{0}$ (m^3^·mol^−1^)	10^6^·*S*_V_ (m^3^·kg·mol^−2^)	10^6^·*σ* (m^3^·mol^−1^)
0.05 l-cysteine			
288.15	117.52 ± 0.004	0.87 ± 0.010	0.003
298.15	118.85 ± 0.006	0.85 ± 0.018	0.004
308.15	119.95 ± 0.006	0.74 ± 0.026	0.006
318.15	120.93 ± 0.005	0.70 ± 0.027	0.006
0.1 l-cysteine			
288.15	117.71 ± 0.009	0.79 ± 0.027	0.007
298.15	118.98 ± 0.007	0.74 ± 0.021	0.005
308.15	120.00 ± 0.007	0.70 ± 0.014	0.003
318.15	121.00 ± 0.008	0.66 ± 0.026	0.006
0.2 l-cysteine			
288.15	117.84 ± 0.005	0.74 ± 0.029	0.007
298.15	119.08 ± 0.005	0.69 ± 0.021	0.004
308.15	120.14 ± 0.003	0.67 ± 0.015	0.003
318.15	121.09 ± 0.005	0.62 ± 0.016	0.003
0.3 l-cysteine			
288.15	117.99 ± 0.005	0.73 ± 0.024	0.005
298.15	119.24 ± 0.003	0.61 ± 0.017	0.003
308.15	120.29 ± 0.005	0.61 ± 0.021	0.005
318.15	121.23 ± 0.008	0.57 ± 0.015	0.004

**Table 8:** The coefficients of Eq. 2 with the corresponding residual standard deviations $\sigma$ for d-sorbitol in l-histidine aqueous solution at temperature *T* = 288.15, 298.15, 308.15 and 318.15 K

*T*/K	10^6^·$\phi_{\text{V}}^{0}$ (m^3^·mol^−1^)	10^6^*S*_V_ (m^3^·kg·mol^−2^)	10^6^*σ* (m^3^·mol^−1^)
0.05 l-histidine			
288.15	117.57 ± 0.007	0.86 ± 0.019	0.003
298.15	118.87 ± 0.006	0.71 ± 0.021	0.005
308.15	119.97 ± 0.004	0.64 ± 0.018	0.004
318.15	120.93 ± 0.004	0.46 ± 0.015	0.003
0.1 l-histidine			
288.15	117.77 ± 0.005	0.73 ± 0.029	0.006
298.15	119.02 ± 0.006	0.62 ± 0.015	0.003
308.15	120.07 ± 0.004	0.59 ± 0.022	0.005
318.15	121.05 ± 0.005	0.51 ± 0.017	0.003
0.2 l-histidine			
288.15	117.99 ± 0.005	0.52 ± 0.015	0.004
298.15	119.22 ± 0.004	0.50 ± 0.014	0.003
308.15	120.27 ± 0.005	0.49 ± 0.016	0.004
318.15	121.20 ± 0.006	0.46 ± 0.018	0.004

**Table 9:** The coefficients of Eq. 2 with the corresponding residual standard deviations $\sigma$ for d-sorbitol in NaCl aqueous solution at temperature *T* = 288.15, 298.15, 308.15 and 318.15 K

*T*/K	10^6^·$\phi_{\text{V}}^{0}$ (m^3^·mol^−1^)	10^6^·*S*_V_ (m^3^·kg·mol^−2^)	10^6^*σ* (m^3^·mol^−1^)
0.05 NaCl			
288.15	117.61 ± 0.008	0.84 ± 0.024	0.006
298.15	118.92 ± 0.006	0.73 ± 0.016	0.004
308.15	120.03 ± 0.006	0.58 ± 0.020	0.004
318.15	121.01 ± 0.005	0.52 ± 0.012	0.003
0.1 NaCl			
288.15	117.78 ± 0.005	0.52 ± 0.021	0.005
298.15	119.05 ± 0.004	0.50 ± 0.012	0.003
308.15	120.11 ± 0.005	0.49 ± 0.018	0.004
318.15	121.08 ± 0.005	0.39 ± 0.017	0.004
0.2 NaCl			
288.15	118.09 ± 0.006	0.48 ± 0.015	0.004
298.15	119.28 ± 0.005	0.43 ± 0.016	0.004
308.15	120.32 ± 0.005	0.40 ± 0.016	0.004
318.15	121.27 ± 0.006	0.38 ± 0.015	0.004
0.3 NaCl			
288.15	118.32 ± 0.004	0.43 ± 0.010	0.002
298.15	119.51 ± 0.007	0.38 ± 0.010	0.003
308.15	120.52 ± 0.004	0.36 ± 0.015	0.003
318.15	121.44 ± 0.007	0.32 ± 0.015	0.003

As can be seen from Tables 2, 3, 4 and 5 and from Fig. 2, the apparent molar volume increases with increasing molality of sugar alcohol and also with the amount of co-solute and with temperature. Irrespective of temperature and molality of amino acid or NaCl, the concentration dependence of the apparent molar volume of d-sorbitol is very small and therefore the standard deviations of the experimental slope *S*_V_ are relatively high. However, a decrease of *S*_V_ with temperature and co–solute concentration can be observed. The results indicate that increasing temperature and increasing molality of amino acid or NaCl make the solute–solute interactions weaker. Additionally, the values of the experimental slope for the same molality of co-solute and the same temperature follow the following order:

$${\textsc {l}}{\text{-alanine}} > {\textsc{l}}{\text{-cysteine}} > {\textsc{l}}{\text{-histidine}} > {\text{sodium chloride}}$$

indicating that solute–solute interactions are weakest in the (d-sorbitol + NaCl + water) ternary system.

An examination of the data also reveals that the limiting apparent molar volumes of d- sorbitol are positive and increase with increasing molality of amino acid or sodium chloride, as well as with increasing temperature. The limiting apparent molar expansibilities $\left( {{{\partial \phi_{\text{V}}^{0} } \mathord{\left/ {\vphantom {{\partial \phi_{\text{V}}^{0} } {\partial T}}} \right. \kern-0pt} {\partial T}}} \right)_{p}$ and their second derivatives $\left( {{{\partial^{2} \phi_{\text{V}}^{0} } \mathord{\left/ {\vphantom {{\partial^{2} \phi_{\text{V}}^{0} } {\partial T^{2} }}} \right. \kern-0pt} {\partial T^{2} }}} \right)_{p}$, presented in Table 10, were calculated by fitting the $\phi_{\text{V}}^{0}$ data, as a function of temperature, into the following equation:

3 $$\phi_{\text{V}}^{0} = A_{0} + A_{1} T + A_{2} T^{2}$$

using the least-squares method, where *A*_0_, *A*_1_ and *A*_2_ are constants. As is seen, the values of the limiting apparent molar expansibilities for all the systems studied are positive. This suggests that the temperature increase causes the liberation of solvent molecules from the solvation layer of d-sorbitol, thereby increasing the total volume of the system. Moreover, the fact that the limiting apparent molar expansibility values decrease with rising temperature indicates that at higher temperatures this effect is weaker.

**Table 10:** Limiting apparent molar expansibilities and the second-order derivatives of $\phi_{\text{V}}$ for d-sorbitol in L-aniline, l-cysteine, l-histidine and NaCl aqueous solution at temperatures *T* = 288.15, 298.15, 308.15 and 318.15 K

*m*_b_ (mol·kg^−1^)	10^6^· $\left( {{{\partial \phi_{\text{V}}^{0} } \mathord{\left/ {\vphantom {{\partial \phi_{\text{V}}^{0} } {\partial T}}} \right. \kern-0pt} {\partial T}}} \right)_{p}$ (m^3^·K^−1^·mol^−1^)				10^6^·*σ* (m^3^·K^−1^·mol^−1^)	10^6^· $\left( {{{\partial^{2} \phi_{\text{V}}^{0} } \mathord{\left/ {\vphantom {{\partial^{2} \phi_{\text{V}}^{0} } {\partial T^{2} }}} \right. \kern-0pt} {\partial T^{2} }}} \right)_{p}$ (m^3^·K^−2^·mol^−1^)
*T/*K	288.15	298.15	308.15	318.15		
Water						
0	0.136	0.122	0.109	0.095	0.0064	− 0.0014
l-alanine						
0.05	0.134	0.120	0.107	0.094	0.0024	− 0.0013
0.1	0.136	0.119	0.102	0.086	0.0029	− 0.0017
0.2	0.135	0.117	0.100	0.082	0.0088	− 0.0018
0.3	0.131	0.115	0.100	0.084	0.014	− 0.0016
l-cysteine						
0.05	0.140	0.122	0.104	0.087	0.017	− 0.0018
0.1	0.133	0.117	0.101	0.085	0.011	− 0.0016
0.2	0.130	0.115	0.101	0.086	0.010	− 0.0015
0.3	0.130	0.115	0.100	0.085	0.013	− 0.0015
l-histidine						
0.05	0.137	0.120	0.103	0.087	0.0062	− 0.0017
0.1	0.129	0.116	0.102	0.089	0.016	− 0.0013
0.2	0.129	0.114	0.099	0.085	0.0075	− 0.0015
NaCl						
0.05	0.138	0.121	0.105	0.089	0.010	− 0.0016
0.1	0.132	0.117	0.102	0.087	0.018	− 0.0015
0.2	0.123	0.112	0.100	0.088	0.010	− 0.0012
0.3	0.124	0.110	0.097	0.084	0.015	− 0.0013

The calculated values of the second derivate $\left( {{{\partial^{2} \phi_{\text{V}}^{0} } \mathord{\left/ {\vphantom {{\partial^{2} \phi_{\text{V}}^{0} } {\partial T^{2} }}} \right. \kern-0pt} {\partial T^{2} }}} \right)_{p}$ are negative. Therefore, according to Hepler’s method of examining the sign of the second derivate for solute in terms of the structure-making or structure-breaking nature of the solute in mixed solvent systems, d-sorbitol is a structure breaker in water, as well as in aqueous amino acid and sodium chloride [22].

**Fig. 3:**
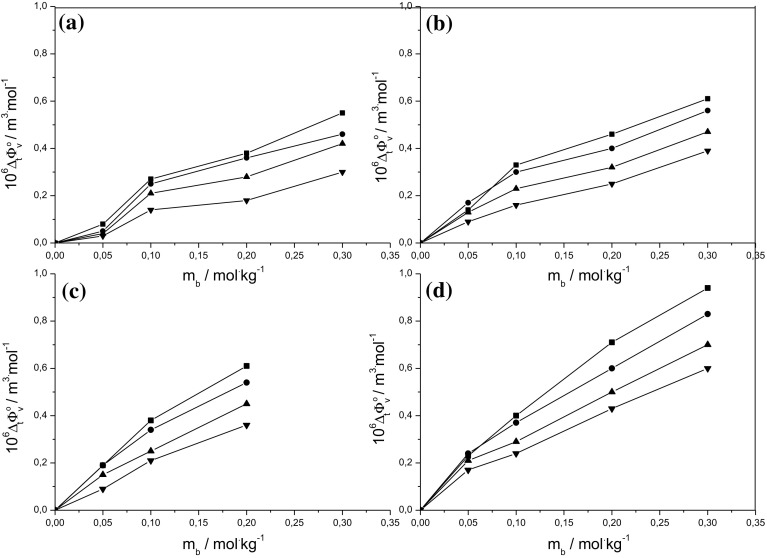
The limiting apparent molar volumes of transfer of d-sorbitol versus molalities of l-alanine (**a**), l-cysteine (**b**), l-histidine (**c**) and NaCl (**d**). Symbols the same as in Fig. 1

Figure 3 presents the limiting apparent molar volumes of transfer of d-sorbitol, $\Delta_{\text{t}} \phi_{\text{V}}^{0}$, versus molalities of l-alanine, l-cysteine, l-histidine and NaCl. The values of $\Delta_{\text{t}} \phi_{\text{V}}^{0}$ from aqueous to aqueous co-solute solutions were calculated as:

4 $$\Delta_{\text{t}} \phi_{\text{V}}^{0} = \phi_{\text{V}}^{0} \left( {{\text{H}}_{2} {\text{O}}\;{ + }\;{\text{co-solute}}} \right) - \phi_{\text{V}}^{0} \left( {{\text{H}}_{2} {\text{O}}} \right)$$

It has been established by Shahidi, Farrell and Edward that the limiting apparent molar volume of a hydrophilic solute in water can be divided into two terms as:

5 $$\phi_{\text{V}}^{0} = V_{\text{int}} - n\sigma$$

where *V*_int_ is the intrinsic molar volume, *σ* is the shrinkage in the volume due to hydrogen bonding between water and the hydrophilic solute and *n* denotes the number of hydrogen bonding sites [23]. Moreover, the intrinsic molar volume *V*_int_ consists of the van der Waals volume and the associated void or empty volume. For dilute solutions, it is reasonable to assume that the intrinsic molar volume is nearly constant with respect to concentration. Thus, the observed positive volume change accompanying the transfer of d-sorbitol from water to aqueous amino acid or NaCl solution may result from a decrease in the shrinkage in volume because of solute co-solute interactions. Additionally, the increasing values of the limiting apparent molar volumes of transfer with molality of NaCl or amino acid reveal that the interactions resulting in the dehydration of the sugar alcohol increase with the concentration of co-solute.

As can be seen from Fig. 3, at all temperatures studied for all systems, the values of the limiting apparent molar volumes of transfer are in the following ascending order:

$${\textsc{l}}{\text{-alanine}}> {\textsc{l}}{\text{-cysteine}}>{\textsc{l}}{\text{-histidine}}> {\text{sodium chloride}}$$

indicating that the strongest solute co-solute interactions occur in the (d-sorbitol + NaCl + water) ternary system.

The interactions between d-sorbitol and l-alanine, l-cysteine, l-histidine and NaCl in aqueous solutions can also be explained on the basis of the co-sphere overlap model [24]. According to the model, when two molecules approach each other, their hydration co-spheres overlap and the physico-chemical properties of the solution, such as limiting apparent molar volume, change. The possible types of interactions between solute and co-solute are:

1. hydrophilic–ionic interactions between hydrophilic groups of solute and ions of co-solute;
2. hydrophobic–ionic interactions between the hydrophobic alkyl chain of solute and ions of co-solute;
3. hydrophilic–hydrophilic interactions between the hydrophilic groups of solute and the hydrophilic groups of co-solute;
4. hydrophobic–hydrophobic interactions between the hydrophobic alkyl chain of solute and the hydrophobic alkyl chain of co-solute;
5. hydrophobic–hydrophilic interactions between the hydrophobic alkyl chain of solute and the hydrophilic groups of co-solute.

It is obvious that in solutions of d-sorbitol in aqueous amino acids all five types of interactions are possible, while in solutions of d-sorbitol in aqueous sodium chloride only two types of interactions, i.e. (1) and (2), can occur. Hydrophilic–ionic interactions and hydrophilic–hydrophilic interactions result in positive values of the limiting apparent molar volumes of transfer, whereas hydrophobic–ionic, hydrophobic–hydrophobic and hydrophobic–hydrophilic interactions result in negative $\Delta_{\text{t}} \phi_{\text{V}}^{0}$ values. The observed positive values of $\Delta_{\text{t}} \phi_{\text{V}}^{0}$ suggest that for d-sorbitol in aqueous NaCl interactions of type (1) and for d-sorbitol in aqueous amino acids interactions of types (1) and (3) are predominant.

**Fig. 4:**
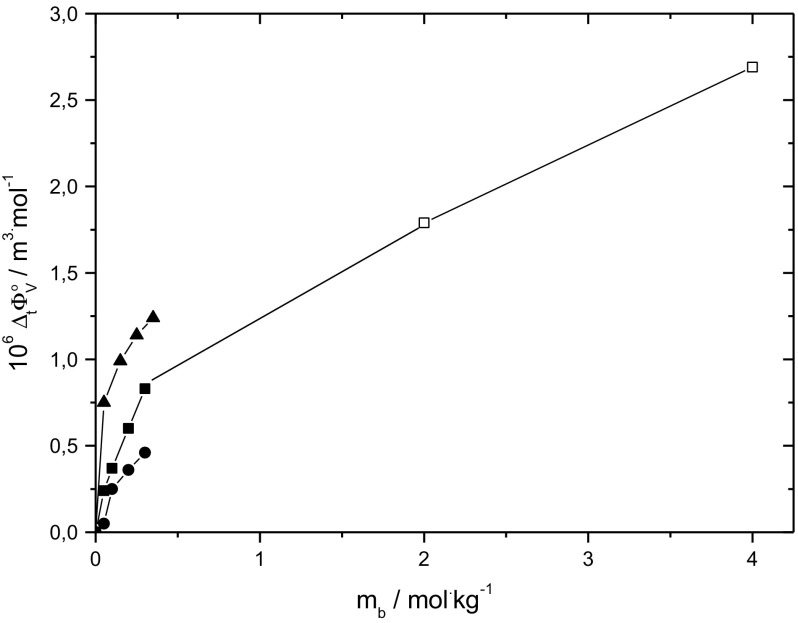
The limiting apparent molar volumes of transfer of d-sorbitol versus molalities of l-alanine (filled circles) and NaCl (filled squares) together with the literature data for d-sorbitol versus molalities of l-glycine (filled triangle) and NaCl (open squares) at temperature 298.15 K [18, 21]

Figure 4 presents the limiting apparent molar volumes of transfer for d-sorbitol versus molalities of l-alanine and NaCl at 298.15 K together with the literature data for d-sorbitol in aqueous l-glycine and sodium chloride [18, 21]. For clarity, the experimental values of $\Delta_{\text{t}} \phi_{\text{V}}^{0}$ for d-sorbitol in aqueous l-cysteine and L- histidine solutions are not shown. As is seen from Figs. 3 and 4, d-sorbitol displays highest positive values of the limiting apparent molar volumes of transfer in aqueous l-glycine, the smallest amino acid studied, while lowest values of $\Delta_{\text{t}} \phi_{\text{V}}^{0}$ are observed in l-alanine, the most hydrophobic amino acid studied. Comparison of the limiting apparent molar volumes of transfer of d-sorbitol between amino acids of the same alkyl chain length, i.e. l-alanine, l-cysteine and l-histidine, indicates that $\Delta_{\text{t}} \phi_{\text{V}}^{0}$ is determined by the hydrophilic residues of the amino acid. According to the hydrophobicity scale of amino acids proposed by Kapcha and Rossky, l-histidine is more hydrophilic than l-cysteine. This results in stronger hydrophilic–ionic and hydrophilic–hydrophilic interactions between d-sorbitol and l-histidine than those between d-sorbitol and l-cysteine, leading to more positive values of the limiting apparent molar volumes of transfer [25].

As mentioned above, the experimental data on $\Delta_{\text{t}} \phi_{\text{V}}^{0}$ for d-sorbitol in aqueous sodium chloride are consistent with that in literature. Moreover, the observed values of the limiting apparent molar volumes of transfer for that system are lower than those for (d-sorbitol + l-glycine + water). This is clearly the result of the strong hydrophilic–hydrophilic interactions between d-sorbitol and l-glycine which do not take place between sugar alcohols and NaCl.

In line with the proposition of Friedman and Krishnan, according to which the thermodynamic transfer properties of solutes in dilute aqueous solution are determined by solute and co-solute interactions without the consideration of solute–solute interactions, and in conformity with the McMillan–Mayer theory, the limiting apparent molar volume of transfer can be expressed as:

6 $$\Delta_{\text{t}} \phi_{\text{V}}^{0} = 2V_{\text{ab}} m_{\text{b}} + 3V_{\text{abb}} m_{\text{b}}^{2}$$

where *V*_ab_ and *V*_abb_ are the pairwise and triplet interaction coefficients, respectively, and *m*_b_ is the molality of the co-solute [26, 27]. Table 11 presents the values of the volumetric interaction coefficients of d-sorbitol in aqueous L-aniline, l-cysteine, L- histidine and NaCl solutions obtained in the present study. As is seen, in all cases the pairwise interaction coefficients are positive while the triplet interaction coefficients are negative. In general, the magnitude of volume interaction coefficients decreases with rising temperature. The fact that the pairwise interaction coefficients are positive and larger in magnitude than the triplet interaction coefficients suggests that the volumetric properties of the studied solutions are determined by the strong interactions between d-sorbitol and the amino acids or sodium chloride and that these interactions dominate over triplet interactions. Moreover, the decrease of volume interaction coefficients with rising temperature indicates that pair-wise interactions, as well as triplet interactions, become weaker at higher temperatures.

**Table 11:** Pair, *V*_AB_ and triplet, *V*_ABB_ interaction coefficients of d-sorbitol in L-aniline, l-cysteine, L- histidine and NaCl aqueous solution at temperature *T* = 288.15, 298.15, 308.15 and 318.15 K

Compound	10^6.^*V*_ab_ (m^3^·kg·mol^−2^)	10^6.^*V*_abb_ (m^3^·kg^2^·mol^−3^)	10^6.^*V*_ab_ (m^3^·kg·mol^−2^)	10^6.^*V*_abb_ (m^3^·kg^2^·mol^−3^)	10^6.^*V*_ab_ (m^3^·kg·mol^−2^)	10^6.^*V*_abb_ (m^3^·kg^2^·mol^−3^)	10^6.^*V*_ab_ (m^3^·kg·mol^−2^)	10^6.^*V*_abb_ (m^3^·kg^2^·mol^−3^)
*T*/K	288.15		298.15		308.15		318.15	
l-alanine	1.2 ± 0.13	− 0.7 ± 0.29	1.2 ± 0.13	− 0.9 ± 0.37	0.8 ± 0.17	− 0.4 ± 0.31	0.5 ± 0.12	− 0.1 ± 0.22
l-cysteine	1.7 ± 0.10	− 1.5 ± 0.25	1.5 ± 0.17	− 1.4 ± 0.33	1.1 ± 0.14	− 0.9 ± 0.25	0.78 ± 0.060	− 0.3 ± 0.27
l-histidine	2.18 ± 0.043	− 2.2 ± 0.20	2.06 ± 0.010	− 2.38 ± 0.025	1.47 ± 0.044	− 1.15 ± 0.022	1.09 ± 0.052	− 0.6 ± 0.20
NaCl	2.25 ± 0.034	− 1.53 ± 0.085	2.0 ± 0.16	− 1.5 ± 0.30	1.6 ± 0.16	− 1.1 ± 0.29	1.37 ± 0.077	− 0.8 ± 0.25

### Acoustic Properties

The experimental values of molality, sound velocity and apparent molar compressibility at 298.15 K of the solutions of d-sorbitol in aqueous l-alanine, l-cysteine, L- histidine and NaCl are reported in Tables 12, 13, 14, and 15. As is seen, the values of sound velocity increase with increasing molality of sugar alcohol, both in water and in the aqueous solutions of amino acids or sodium chloride. This suggests that as the molality increases, intermolecular hydrogen bonds between d-sorbitol and water and intramolecular hydrogen bonds within d-sorbitol molecules themselves become stronger, resulting in a more ordered solution. Moreover, for solutions with the same molality of d-sorbitol, an increase in sound velocity with growing concentration of co-solute is also observed. This suggests that association among the molecules of the solution becomes stronger as the amount of amino acid or NaCl in solution increases.

**Table 12:** The sound velocities and apparent molar compressibilities of d-sorbitol in l-alanine aqueous solutions at temperature *T* = 298.15 K

*m* (mol·kg^−1^)	*u* (m·s^−1^)	10^15.^*ϕ*_KS_ (m^5^·mol^−1^·N^−1^)	*m* (mol·kg^−1^)	*u* (m·s^−1^)	10^15.^*ϕ*_KS_ (m^5^·mol^−1^·N^−1^)	*m* (mol·kg^−1^)	*u* (m·s^−1^)	10^15.^*ϕ*_KS_ (m^5^·mol^−1^·N^−1^)	*m* (mol·kg^−1^)	*u* (m·s^−1^)	10^15.^*ϕ*_K_ (m^5^·mol^−1^·N^−1^)	*m* (mol·kg^−1^)	*u* (m·s^−1^)	10^15.^*ϕ*_KS_ (m^5^·mol^−1^·N^−1^)
Water			0.05 mol·kg^−1^			0.1 mol·kg^−1^			0.2 mol·kg^−1^			0.3 mol·kg^−1^		
0.0000	1496.69	–	0.0000	1499.78	–	0.0000	1503.43	–	0.0000	1509.40	–	0.0000	1515.00	–
0.04020	1499.30	− 13.9	0.04812	1502.94	− 13.8	0.03207	1505.54	− 13.6	0.04673	1512.50	− 13.0	0.04969	1518.33	− 12.7
0.06324	1500.80	− 13.6	0.06101	1503.79	− 13.7	0.05503	1507.05	− 13.3	0.05916	1513.34	− 13.0	0.06298	1519.19	− 12.2
0.08443	1502.20	− 13.4	0.07938	1504.99	− 13.3	0.07659	1508.42	− 12.6	0.07854	1514.60	− 12.5	0.07810	1520.19	− 12.0
0.09748	1503.05	− 13.2	0.09376	1505.92	− 13.0	0.09114	1509.40	− 12.6	0.09352	1515.63	− 12.5	0.09708	1521.45	− 11.7
0.1273	1505.02	− 12.9	0.1207	1507.71	− 12.7	0.1165	1511.08	− 12.3	0.1198	1517.38	− 12.1	0.1264	1523.42	− 11.4
0.1472	1506.25	− 12.3	0.1581	1510.22	− 12.4	0.1514	1513.36	− 11.7	0.1560	1519.80	− 11.6	0.1621	1525.74	− 10.7
0.1741	1508.10	− 12.3	0.1722	1511.16	− 12.2	0.1655	1514.30	− 11.6	0.1661	1520.43	− 11.4	0.1747	1526.61	− 10.7
0.1986	1509.66	− 11.8	0.1922	1512.50	− 12.0	0.1845	1515.55	− 11.3	0.1886	1521.97	− 11.2	0.1978	1528.06	− 10.1
0.2368	1512.22	− 11.4	0.2250	1514.68	− 11.6	0.2168	1517.65	− 10.8	0.2203	1524.00	− 10.5	0.2320	1530.39	− 9.8
0.2782	1515.07	− 11.1	0.2671	1517.52	− 11.1	0.2571	1520.33	− 10.3	0.2629	1526.90	− 10.1	0.2764	1533.32	− 9.2
0.3167	1517.65	− 10.6	0.3042	1519.96	− 10.5	0.2932	1522.64	− 9.7	0.2997	1529.22	− 9.4	0.3149	1535.96	− 8.9

Standard uncertainties u are u(*T*) = 0.01 K, u(*u*) = 0.15 m·s^−1^, standard uncertainty of molality u(*m*) = 0.001 mol·kg^−1^, and standard uncertainty of experimental pressure u(*p*) = 10 kPa

**Table 13:** The sound velocities and apparent molar compressibilities of d-sorbitol in l-cysteine aqueous solutions at temperature *T* = 298.15 K

*m* (mol·kg^−1^)	*u* (m·s^−1^)	10^15.^*ϕ*_KS_ (m^5^·mol^−1^·N^−1^)	*m* (mol·kg^−1^)	*u* (m·s^−1^)	10^15.^*ϕ*_KS_ (m^5^·mol^−1^·N^−1^)	*m* (mol·kg^−1^)	*u* (m·s^−1^)	10^15.^*ϕ*_KS_ (m^5^·mol^−1^·N^−1^)	*m* (mol·kg^−1^)	*u* (m·s^−1^)	10^15.^*ϕ*_KS_ (m^5^·mol^−1^·N^−1^)
0.05 mol·kg^−1^			0.1 mol·kg^−1^			0.2 mol·kg^−1^			0.3 mol·kg^−1^		
0.0000	1500.34	–	0.0000	1504.28	–	0.0000	1511.00	–	0.0000	1517.94	–
0.06327	1504.48	− 13.2	0.05122	1507.60	− 12.4	0.06330	1515.20	− 12.2	0.04672	1521.10	− 12.2
0.07674	1505.35	− 12.9	0.06342	1508.38	− 12.2	0.08324	1516.50	− 11.8	0.05997	1521.97	− 11.7
0.09873	1506.83	− 12.9	0.08443	1509.76	− 12.0	0.1016	1517.74	− 11.6	0.07901	1523.23	− 11.4
0.1259	1508.62	− 12.5	0.1004	1510.79	− 11.8	0.1205	1519.01	− 11.5	0.09337	1524.23	− 11.4
0.1630	1511.08	− 12.0	0.1252	1512.40	− 11.4	0.1574	1521.39	− 10.7	0.1202	1526.05	− 11.1
0.1783	1512.03	− 11.6	0.1621	1514.79	− 10.9	0.1928	1523.71	− 10.2	0.1550	1528.45	− 10.8
0.1993	1513.36	− 11.2	0.1762	1515.67	− 10.6	0.2258	1526.02	− 10.1	0.1693	1529.38	− 10.5
0.2327	1515.55	− 10.8	0.2009	1517.27	− 10.3	0.2680	1528.64	− 9.2	0.1891	1530.69	− 10.2
0.2782	1518.52	− 10.1	0.2349	1519.38	− 9.6	0.3065	1531.27	− 8.8	0.2224	1533.02	− 9.9
0.3166	1521.11	− 9.8	0.2836	1522.55	− 9.0				0.2623	1535.67	− 9.3
			0.3195	1524.87	− 8.6				0.3013	1538.30	− 8.8

Standard uncertainties u are u(*T*) = 0.01 K, u(*u*) = 0.15 m·s^−1^, standard uncertainty of molality u(*m*) = 0.001 mol·kg^−1^, and standard uncertainty of experimental pressure u(*p*) = 10 kPa

**Table 14:** The sound velocities and apparent molar compressibilities of d-sorbitol in l-histidine aqueous solutions at temperature *T* = 298.15 K

*m* (mol·kg^−1^)	*u* (m·s^−1^)	10^15.^*ϕ*_KS_ (m^5^·mol^−1^·N^−1^)	*m* (mol·kg^−1^)	*u* (m·s^−1^)	10^15.^*ϕ*_KS_ (m^5^·mol^−1^·N^−1^)	*m* (mol·kg^−1^)	*u* (m·s^−1^)	10^15.^*ϕ*_KS_ (m^5^·mol^−1^·N^−1^)
0.05 mol·kg^−1^			0.1 mol·kg^−1^			0.2 mol·kg^−1^		
0.0000	1500.89	–	0.0000	1505.46	–	0.0000	1513.36	–
0.05323	1504.39	− 13.5	0.04773	1508.55	− 12.3	0.04743	1516.50	− 11.9
0.06586	1505.22	− 13.3	0.06064	1509.38	− 12.0	0.06299	1517.52	− 11.6
0.07847	1506.05	− 13.1	0.07851	1510.56	− 12.0	0.08371	1518.90	− 11.4
0.09625	1507.20	− 12.7	0.09436	1511.60	− 11.8	0.09852	1519.85	− 11.1
0.1229	1508.96	− 12.4	0.1271	1513.74	− 11.4	0.1254	1521.63	− 10.7
0.1551	1511.08	− 12.0	0.1682	1516.41	− 10.8	0.1636	1524.19	− 10.4
0.1705	1512.12	− 11.9	0.1910	1517.94	− 10.6	0.1784	1525.16	− 10.1
0.1935	1513.65	− 11.6	0.2240	1520.09	− 10.2	0.1992	1526.51	− 9.8
0.2235	1515.64	− 11.2	0.2673	1522.87	− 9.5	0.2339	1528.84	− 9.4
0.2630	1518.23	− 10.6	0.3038	1525.16	− 8.9	0.2783	1531.86	− 9.0
0.3063	1521.11	− 10.1				0.3175	1534.49	− 8.6

Standard uncertainties u are u(*T*) = 0.01 K, u(*u*) = 0.15 m·s^−1^, standard uncertainty of molality u(*m*) = 0.001 mol·kg^−1^, and standard uncertainty of experimental pressure u(*p*) = 10 kPa

**Table 15:** The sound velocities and apparent molar compressibilities of d-sorbitol in NaCl aqueous solutions at temperature *T* = 298.15 K

*m* (mol·kg^−1^)	*u* (m·s^−1^)	10^15.^*ϕ*_KS_ (m^5^·mol^−1^·N^−1^)	*m* (mol·kg^−1^)	*u* (m·s^−1^)	10^15.^*ϕ*_KS_ (m^5^·mol^−1^·N^−1^)	*m* (mol·kg^−1^)	*u* (m·s^−1^)	10^15.^*ϕ*_KS_ (m^5^·mol^−1^·N^−1^)	*m* (mol·kg^−1^)	*u* (m·s^−1^)	10^15.^*ϕ*_KS_ (m^5^·mol^−1^·N^−1^)
0.05 mol·kg^−1^			0.1 mol·kg^−1^			0.2 mol·kg^−1^			0.3 mol·kg^−1^		
0.0000	1500.06	–	0.0000	1502.82	–	0.0000	1508.85	–	0.0000	1514.79	–
0.05128	1503.36	− 12.7	0.04895	1505.99	− 12.5	0.05714	1512.50	− 11.0	0.06543	1519.02	− 10.4
0.06540	1504.28	− 12.6	0.06382	1506.94	− 12.2	0.07229	1513.46	− 10.7	0.08987	1520.62	− 10.2
0.08638	1505.66	− 12.5	0.08004	1508.00	− 12.1	0.09448	1514.90	− 10.6	0.1059	1521.68	− 10.1
0.1002	1506.54	− 12.2	0.09695	1509.09	− 11.8	0.1129	1516.12	− 10.5	0.1407	1524.00	− 9.8
0.1310	1508.55	− 11.9	0.1249	1510.93	− 11.6	0.1454	1518.22	− 10.1	0.1736	1526.22	− 9.6
0.1684	1511.07	− 11.7	0.1578	1513.12	− 11.3	0.1860	1520.88	− 9.7	0.1935	1527.57	− 9.5
0.1844	1512.12	− 11.5	0.1759	1514.32	− 11.1	0.2041	1522.11	− 9.6	0.2162	1529.13	− 9.3
0.2060	1513.54	− 11.2	0.1956	1515.65	− 11.0	0.2276	1523.71	− 9.5	0.2511	1531.56	− 9.1
0.2412	1515.93	− 10.9	0.2301	1518.00	− 10.7	0.2675	1526.32	− 9.0	0.2983	1534.79	− 8.7
0.2876	1518.94	− 10.2	0.2616	1520.19	− 10.6	0.3184	1529.81	− 8.7	0.3448	1538.14	− 8.5
0.3277	1521.68	− 9.8	0.3121	1523.71	− 10.2	0.3635	1533.03	− 8.5			

Standard uncertainties u are u(*T*) = 0.01 K, u(*u*) = 0.15 m·s^−1^, standard uncertainty of molality u(*m*) = 0.001 mol·kg^−1^, and standard uncertainty of experimental pressure u(*p*) = 10 kPa

The apparent molar compressibilities, *ϕ*_KS_, of d-sorbitol in the ternary systems were calculated from the densities and the sound velocities of the solutions according to the equation:

7 $$\phi_{\text{KS}} = \frac{{\left( {d_{0} K_{\text{S}} - dK_{\text{S}}^{0} } \right)}}{{mdd_{0} }} + \frac{{M_{2} K_{\text{S}} }}{d}$$

where *K*_S_ and $K_{\text{S}}^{0}$ denote isentropic compressibilities of the solution and solvent, respectively.

The isentropic compressibility *K*_S_ was obtained from density and sound velocity values using the Laplace equation:

8 $$K_{\text{S}} = \frac{1}{{u^{2} d}}$$

As can be observed in Tables 12 and 15, the values of the apparent molar compressibilities are negative for all the systems studied. This indicates a decrease in compressibility of the solution compared with the pure solvent, as a result of strong solute and solvent interactions.

The apparent molar compressibilities were found to increase linearly with the molality of d-sorbitol. Thus, the apparent molar isentropic compressibilities at infinite dilution were obtained by using the method of linear regression of the following relation:

9 $$\phi_{\text{KS}} = \phi_{\text{KS}}^{0} + S_{\text{K}} m$$

Table 16 presents the parameters of Eq. 9 together with the value of the limiting apparent molar compressibility of d-sorbitol in water obtained by Banipal et al. [19]. As is seen, the data from the present study is consistent with that from literature. To the best of my knowledge, no literature data on the compressibility data of d-sorbitol in aqueous amino acid or NaCl solutions is available for comparison purposes.

**Table 16:** The coefficients of Eq. 9 with the corresponding residual standard deviations $\sigma$ for d-sorbitol in L-aniline, l-cysteine, l-histidine and NaCl aqueous solution at *T* = 298.15 K

m_b_ (mol·kg^−1^)	10^15^ *ϕ*_K_ (m^5^·N^−1^·mol^−1^)	10^15^ *S*_k_ (m^5^·kg·N^−1^·mol^−2^)	10^15^ σ (m^5^·N^−1^·mol^−1^)
Water			
0	− 14.3 ± 0.11 (− 14.29^a^)	12.0 ± 0.48	0.13
l-aniline			
0.05	− 14.3 ± 0.09	12.4 ± 0.30	0.08
0.1	− 13.9 ± 0.09	14.2 ± 0.47	0.12
0.2	− 13.7 ± 0.12	10.5 ± 0.42	0.11
0.3	− 13.1 ± 0.15	14.2 ± 0.51	0.14
l-cysteine			
0.05	− 14.2 ± 0.14	14.2 ± 0.53	0.13
0.1	− 13.2 ± 0.05	14.6 ± 0.28	0.15
0.2	− 13.0 ± 0.04	13.9 ± 0.57	0.14
0.3	− 12.6 ± 0.13	12.3 ± 0.47	0.13
l-histidine			
0.05	− 14.1 ± 0.06	13.2 ± 0.24	0.06
0.1	− 13.0 ± 0.12	12.8 ± 0.44	0.11
0.2	− 12.4 ± 0.10	12.2 ± 0.37	0.10
NaCl			
0.05	− 13.4 ± 0.10	10.6 ± 0.30	0.09
0.1	− 12.7 ± 0.13	8.6 ± 0.40	0.11
0.2	− 11.4 ± 0.09	8.4 ± 0.30	0.09
0.3	− 10.8 ± 0.03	6.8 ± 0.14	0.04

^a^Ref. [19]

The values of the limiting apparent molar compressibilities of d-sorbitol increase (become less negative) with the increase in molality of co-solute. Obviously, this is the result of the fact that when sugar alcohol is dissolved in aqueous amino acid or salt solution, interactions between co-solute and water and between water molecules themselves are disrupted and interactions between solute and co-solute and between solute and water are formed. Thus, with increasing concentration of co-solute, more water molecules are released into the bulk solution and the solution becomes more compressible.

The obtained values of $\phi_{\text{KS}}^{0}$ were used to calculate the limiting apparent molar compressibilities of transfer of d-sorbitol from aqueous to aqueous amino acid or sodium chloride solutions according to the equation:

10 $$\Delta_{\text{t}} \phi_{\text{KS}}^{0} = \phi_{\text{KS}}^{0} \left( {{\text{H}}_{2} {\text{O}}\; + \;{\text{co-solute}}} \right) - \phi_{\text{KS}}^{0} \left( {{\text{H}}_{2} {\text{O}}} \right)$$

**Fig. 5:**
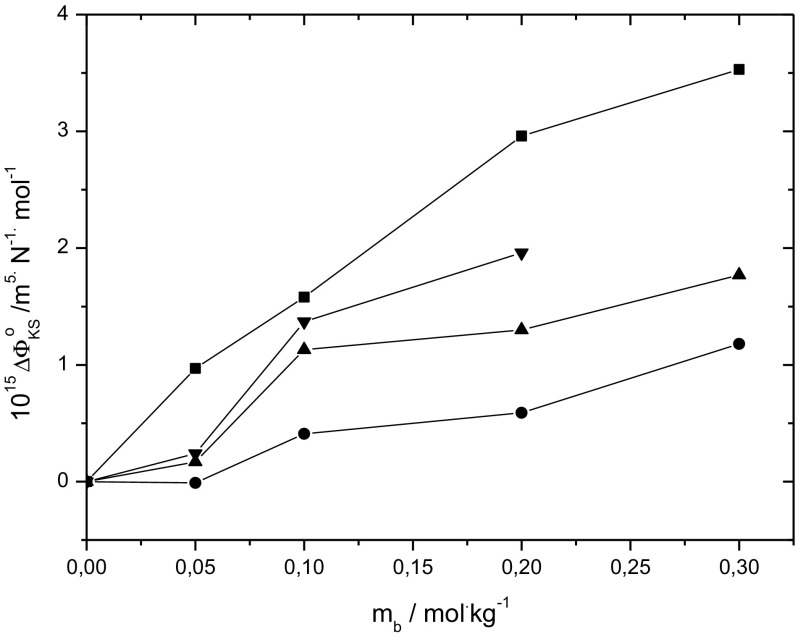
The limiting apparent molar compressibilities of transfer of d-sorbitol versus molalities of l-alanine (filled circles), l-cysteine (filled squares), l-histidine (filled inverted triangle) and NaCl (filled square) at 298.15 K

Figure 5 presents the limiting apparent molar compressibilities of transfer of d-sorbitol versus molalities of l-alanine, l-cysteine, L- histidine and NaCl. It can be seen that the $\Delta_{\text{t}} \phi_{\text{KS}}^{0}$ values are positive, they increase with the concentration of the co-solute and they may be arranged in the same ascending order as $\Delta_{\text{t}} \phi_{\text{V}}^{0}$ values, i.e. l-alanine < l-cysteine < l-histidine < sodium chloride. The results obtained from acoustic studies are in accordance with the results from volumetric studies discussed earlier. They support the conclusion that the hydrophilic–ionic and hydrophilic–hydrophilic interactions in solutions of d-sorbitol in aqueous amino acids are predominant, while the properties of solutions of d-sorbitol in aqueous sodium chloride are determined by hydrophilic–ionic interactions. Moreover, the absence of hydrophobic–hydrophobic and hydrophilic–hydrophobic interactions in the ternary system (d-sorbitol + NaCl + water) results in more positive values of the limiting apparent molar properties of transfer.

## Conclusions

The limiting apparent molar volumes and the limiting apparent molar compressibilities of transfer have been determined for the transfer of d-sorbitol from water to aqueous solutions of l-alanine, l-cysteine, l-histidine and NaCl. Positive $\Delta_{\text{t}} \phi_{\text{V}}^{0}$ and $\Delta_{\text{t}} \phi_{\text{KS}}^{0}$ values have been obtained in all cases, and their magnitude increases with increasing concentration of co-solute, indicating the domination of hydrophilic–ionic interactions in solutions of d-sorbitol in aqueous sodium chloride and the co-domination of hydrophilic–ionic and hydrophilic–hydrophilic interactions in solutions of d-sorbitol in aqueous amino acids. As the obtained values of the limiting apparent molar expansibilities are positive and decrease as the temperature rises, these dominating effects also increase with rising temperature.

The fact that the highest values of the limiting apparent molar volumes and the limiting apparent molar compressibilities were obtained for the (d-sorbitol + NaCl + water) ternary system indicates that interactions between solute and co-solute in this system are stronger than in (d-sorbitol + amino acid + water) mixtures. Moreover, the order of $\Delta_{\text{t}} \phi_{\text{V}}^{0}$ and $\Delta_{\text{t}} \phi_{\text{KS}}^{0}$ values obtained for d-sorbitol in aqueous solutions of l-alanine, l-cysteine and l-histidine, i.e. three amino acids with the same alkyl chain length, strongly suggests that the strength of alcohol sugar and amino acid interactions is determined by the hydrophobicity of amino acid.

The fact that the pairwise interaction coefficients are positive and of greater magnitude than the triplet interaction coefficients supports the conclusion that the properties of the studied solutions are determined by the strong interactions between d-sorbitol and amino acids or sodium chloride.
